# Atypical Presentation of Oronasal Herpes Simplex Virus (HSV) in an Immunocompromised Patient: A Case Report and Literature Review

**DOI:** 10.7759/cureus.69336

**Published:** 2024-09-13

**Authors:** Luluh Alsughayer, Reem Alamri, Reema Alhadlq, Ahmed Alhussien, Ahmed A Al-Sayed

**Affiliations:** 1 Department of Otolaryngology – Head and Neck Surgery, King Saud University, Riyadh, SAU; 2 College of Medicine, King Saud University, Riyadh, SAU

**Keywords:** atypical presentation, herpes simplex virus, immunocompromised, nasal ulcer, oronasal lesion

## Abstract

Herpes simplex virus (HSV) typically presents with characteristic mucocutaneous vesicular lesions. However, atypical manifestations can occur and can be challenging to diagnose, especially in immunocompromised individuals. This case report describes a nine-year-old immunocompromised girl who developed a left nasal vestibular ulcer covered by hemorrhagic crustation and granulation tissue, progressively worsening to a friable exophytic lesion with intact nasal mucosa. The lesion was refractory to local treatment, wide-spectrum antibiotics, and antifungal therapy, hence requiring a biopsy. The diagnosis of HSV infection was confirmed by histopathology, and IV acyclovir successfully treated the initial infection. This case emphasizes the importance of considering HSV infection when evaluating persistent, refractory skin lesions, particularly in immunocompromised patients, which can ensure early diagnosis and appropriate antiviral therapy.

## Introduction

Herpes simplex virus (HSV) is a double-stranded DNA virus with two main types: HSV-1 associated with orolabial lesions and HSV-2 causing genital herpes. The immune system is typically capable of controlling the infection. However, patients with congenital or acquired immunodeficiency experience an increased susceptibility to more severe HSV infection and reactivation. A 2015-2016 study from the Centers for Disease Control and Prevention (CDC) showed a significant difference in the prevalence of the two HSV types, with a prevalence of 47.8% and 11.9% for HSV-1 and HSV-2, respectively [1].

HSV infection can present with pain, swelling, redness, discharge, headache, malaise, and fever, while some people with HSV may be completely asymptomatic [2]. The characteristic appearance of HSV is a cluster of small, tense vesicles on an erythematous base. However, the infection can have an atypical presentation, particularly in immunocompromised individuals [3,4].

There are several methods to diagnose HSV infection, including a swab test from the lesion for viral culture, polymerase chain reaction (PCR), and Tzanck smear, in addition to HSV antibody serum testing. Sinus CT can be used on specific occasions to aid in the differential diagnosis and evaluate mucocutaneous involvement [5].

Management of immunocompetent patients with HSV rarely requires medications, but applying topical antivirals can help manage symptoms. Meanwhile, for more severe HSV infections or immunocompromised patients, hospitalization for IV acyclovir is the primary treatment, potentially followed by oral antivirals [6].

The literature links mechanical trauma to the development of herpes lesions at the trauma site. This was explained by possible traumatic stimulation of the involved nerve, thereby re-activating the virus in the dorsal root ganglion, or by impairing local cutaneous immune barriers [7,8].

In this paper, we present a challenging case of atypical HSV infection in an immunocompromised patient and review the relevant literature. Reporting such cases can alert physicians to HSV as a differential diagnosis even when lesions look different from the classical appearance, thereby preventing delay in treatment and further complications.

## Case presentation

We present a nine-year-old female known case of combined immunodeficiency, global developmental delay, epilepsy, hypothyroidism, and asthma, with a history of plasmablastic lymphoma in remission, previously treated with chemotherapy. The patient was admitted to the pediatric intensive care unit as a case of community-acquired pneumonia and infective endocarditis, complicated by septic shock. She was on nasal cannula oxygenation for five days. The patient developed crusty wound lesions over the left nasal vestibule, initially thought to be secondary to pressure by the nasal cannula. Similar smaller wounds were noted around the lips, likely linked to patients’ behavioral lip biting. The patient had a previous history of HSV infection one year back.

**Figure 1:**
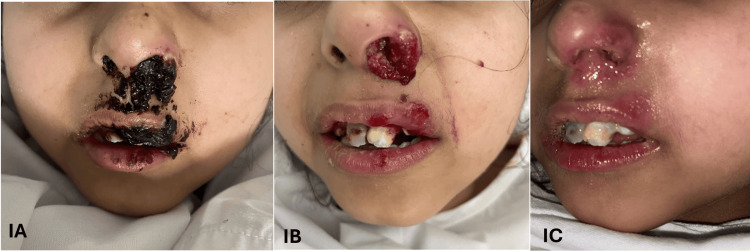
Oronasal herpetic lesions with atypical morphology 1A: Left lip and nasal alar ulcer with granulation tissue almost obstructing the left nasal vestibule. 1B: Exophytic lesion with granulation and surrounding tissue inflammation. 1C: Regression of labio-nasal lesions after systemic antiviral therapy with mild remnant erythema.

**Figure 2:**
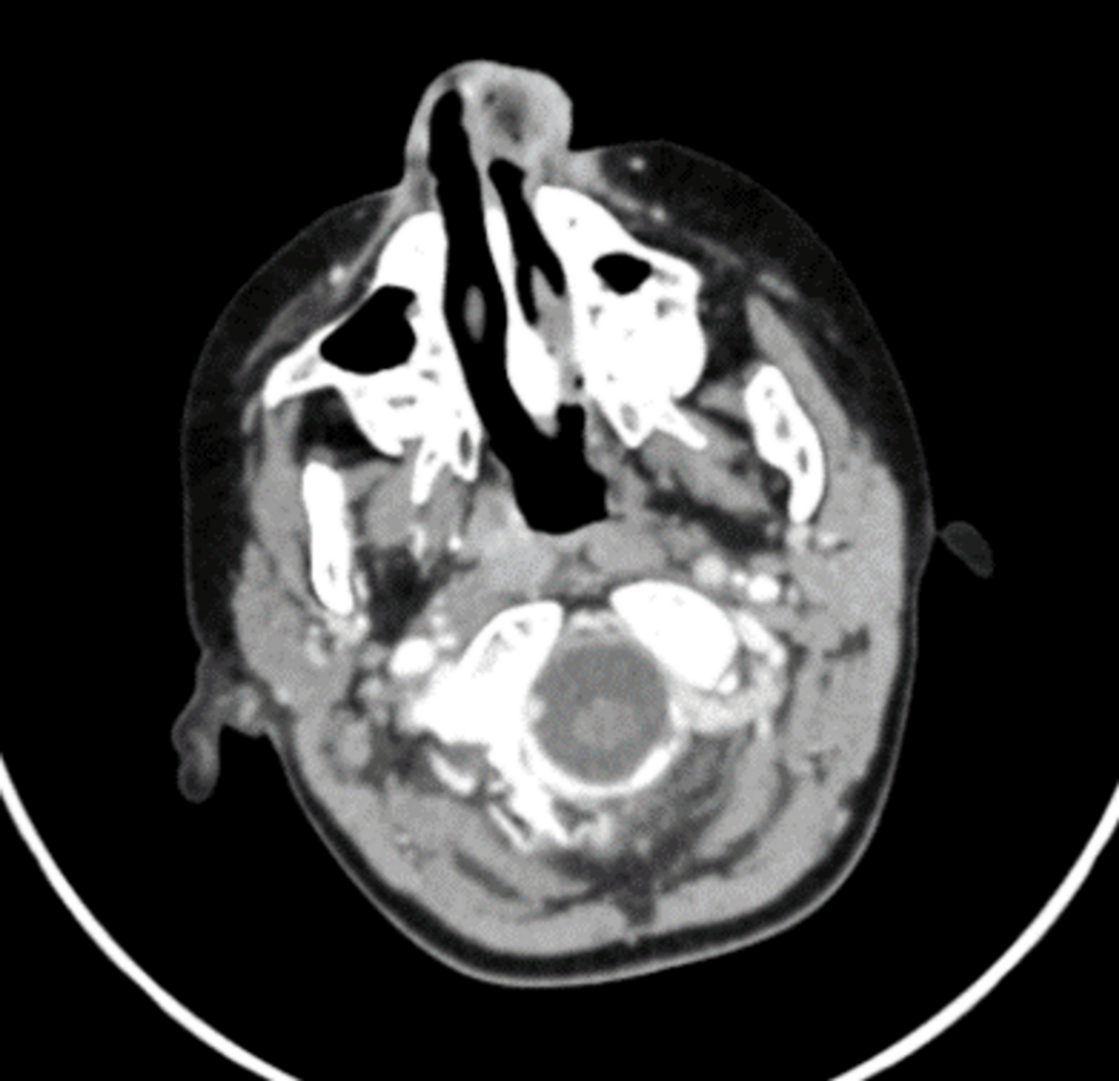
Contrast CT of the nasal herpetic lesion Subcutaneous thickening with inflammatory hyperenhancement at the left nasal alar and tip. CT: computed tomography

Physical examination showed nasal crustation and a left alar ulcer with granulation tissue almost obstructing the left nasal vestibule (Figure 1A). Multiple similar ulcers were noted on the left lip. The nasal scope showed normal nasal mucosa, and the lesion was limited to the vestibule. The nasal wound was rapidly growing, friable to touch, and bleeding with minor manipulation. Bleeding was easily controlled with pressure. The granulation tissue was cauterized with silver nitrate, and daily debridement was done with mupirocin ointment application. The patient was febrile and was hence started on IV antibiotics. After seven days, the lesion persistently grew and did not respond to the ongoing management plan. A swab from the lesion was negative for cytomegalovirus, viral complex, HSV, and acid-fast bacillus (AFB), but PCR testing was positive for Epstein-Barr virus. Contrast CT brain and sinuses showed subcutaneous thickening with inflammatory hyperenhancement at the left nasal alar and tip, no rim enhancement or fluid collection, and no extension to paranasal sinuses or brain (Figure 2).

The patient was still febrile despite IV wide-spectrum antimicrobial coverage with meropenem and vancomycin along with systemic antifungal. Other causes of fever were ruled out using a full septic workup and repeat echo. Meanwhile, she was also receiving monthly IVIG through a port-a-cath. The nasal lesion progressively worsened into an exophytic lesion with surrounding tissue inflammation (Figure 1B). Hence, the patient underwent debridement and biopsy to reach a diagnosis. IV acyclovir was started preliminary. Histopathology results of the left nasal mass biopsy showed fibrinopurulent exudation and necrosis and cells with intranuclear viral inclusion bodies, consistent with herpes virus infection. No malignant cells were noted. Tissue culture results on the patient, including bacterial, fungal, and AFB, were negative.

During 14 days of IV acyclovir, oronasal lesions gradually improved to complete resolution with mild remnant erythema (Figure 1C), and the patient was discharged from the hospital in good condition. Unfortunately, the patient was readmitted to the hospital after a month for cellulitis in her forearm. It was associated with the recurrence of nasal lesions involving the columella. Mother recalls simple trauma with nasal picking. HSV-I PCR came positive. The patient was restarted on acyclovir along with signs of gradual improvement. Consent was secured from the patient’s mother for the purpose of publishing this case report and relevant images.

## Discussion

We presented a case of an immunocompromised child who developed a non-healing nasal lesion. While initial HSV PCR was negative, the diagnosis of herpetic infection was confirmed with histopathology. Despite complete resolution after IV acyclovir treatment, the patient later experienced a recurrence of the nasal lesion with a positive HSV I PCR requiring re-treatment.

The list of differential diagnoses is extensive and includes traumatic ulceration, neoplasm, vasculitis, granulomatous disease, autoimmune disease skin manifestation, and fungal, bacterial, or viral infections. PCR and biopsy are complementary diagnostic modalities for HSV infection. PCR offers non-invasive, rapid diagnosis, high sensitivity, and specificity. However, biopsy remains essential for definitive diagnosis in complex cases, particularly when malignancy or other underlying conditions are suspected [9,10].

The literature reports similar cases of atypical presentation of HSV nasal infections. The presented cases (Table 1) highlight the diverse clinical presentations, including necrotizing rhinitis with hemorrhagic crusting and ulceration and intranasal mass with facial edema. Predisposing factors such as trauma and underlying immunosuppression were evident. Diagnosis often relies on clinical presentation, histopathological findings, and viral culture or PCR. Prompt initiation of antiviral therapy, typically with acyclovir or its analogs, is essential to prevent disease progression and potential complications [3,4,10-13].

**Table 1:** Literature review of cases with atypical herpetic nasal infection M: male, F: female, HSV: herpes simplex virus, EIA: enzyme immunoassay, PCR: polymerase chain reaction, IV: intravenous, TID: three times a day, CT: computer tomography, HIV: human immunodeficiency virus, AFB: acid-fast bacillus, BID: two times a day, MRI: magnetic resonance imaging

Study	Age	Gender	Immune status	History of trauma	Clinical presentation	Diagnosis	Treatment
Powell and Almeyda 2009 [3]	27	F	Immunocompetent	Yes	Erythema of the nose extending from the radix and both medial canthus to the alar rims.	HSV IgM and IgG antibodies, EIA value for HSV IgG type 1 were positive. HSV 1 DNA detected on PCR of the skin swab.	Broad spectrum IV antibiotics were commenced, 5 mg/kg IV acyclovir TID, topical acyclovir.
Patel et al., 2019 [4]	19	F	Immunocompromised	Yes	Nasal cellulitis and crusting, hemorrhagic crusts, and ulceration on an erythematous base involving the nasal columella, ala, base, and upper lip.	laboratory tests (cultures, HSV1 PCR), imaging (CT scan: mild sinus mucosal thickening but no evidence of erosion or invasion of adjacent fat planes), biopsy (inflamed nasal mucosa), and cultures (no growth).	IV acyclovir, topical mupirocin for 2 weeks.
Patel et al., 2019 [4]	8	M	Immunocompromised	No	Fever, thrombocytopenia, eye swelling, periorbital cellulitis, erythematous, slightly cystic nasal lesion.	CT scan (mild sinus mucosal thickening but no evidence of erosion or invasion of adjacent fat planes), laboratory tests (HSV1 PCR).	Broad-spectrum antibiotics (meropenem, vancomycin, amikacin), IV acyclovir for 1 week.
Chiu et al., 2019 [10]	46	F	Immunocompromised	No	Right nasal congestion, hypertrophied turbinates with nasal polyps, right-sided maxillary facial edema, protruding visible exudative mass in right inferior turbinate.	Laboratory tests (HIV viral load and CD4 count, PCR positive for HSV-1 and HSV-2), CT scan (soft tissue opacity in the nasal cavity), and biopsy (cytopathic effects).	Valacyclovir 1 g TID for one month.
Tamashunas et al., 2022 [11]	37	M	Immunocompetent	Yes	Hemorrhagic crusting, ulceration, and black eschar with peripheral erythema of the tip of the nose.	Shave biopsy: ulceration with spongiosis, serous crust, and a lymphohistiocytic infiltrate. Tissue culture: fungi and AFB -ve, group B *Streptococcus* +ve, PCR: HSV-2 +ve	Vancomycin (total of 7 days) and acyclovir (for 2 days). Discharged with a 10-day course of oral valacyclovir 1 g BID and amoxicillin-clavulanate.
Wadhwa et al., 2023 [12]	22	F	Immunocompetent	No	Painful blister on nose tip, extending to right ala.	Serology (IgM for HSV-1)	Amoxicillin-clavulanic acid IV, mupirocin topical, then Valacyclovir orally 1 g BID for 7 days.
Lee et al., 2022 [13]	28	M	Immunocompromised	No	Necrotic lesions on the nasal septum and columella, nasal obstruction due to excessive crust formation, extensive erythema and hemorrhagic crusts in the nasal area, mucosal ulceration, edema, and pallor within the nasal cavity, ulcerative lesions on the lip and gingiva.	MRI (infiltrative lesions and mucosal edema in the left nasal vestibule and anterior nasal cavity). Biopsy (HSV-1 infection).	Antifungal drugs due to suspected fungal infection, IV acyclovir for 3 weeks then shifted to topical.
Lee et al., 2022 [13]	76	M	Immunocompromised	No	Painful ulcerative lesions in both the oral and nasal cavities, nasal obstruction due to crust formation and mucosal inflammation, extensive erythema and hemorrhagic crusts in the nasal vestibule, mucosal ulceration, edema, and pallor in both the oral and nasal cavities.	Biopsy (HSV-1 infection)	IV acyclovir for 1 week then shifted to oral valacyclovir upon discharge.

This case emphasizes the importance of considering HSV infection in immunocompromised patients, especially with persistent lesions unresponsive to broad-spectrum antibiotics and antifungals. This paper helps to direct attention toward improving clinical judgment and tailored management approaches for immunocompromised children with recurrent or unusual lesions.

## Conclusions

Immunocompromised patients can present with severe atypical herpetic lesions. Presentation is not limited to vesicular lesions but extends to polypoidal exophytic lesions, hemorrhagic crusting and ulceration, necrosis, surrounding edema, and erythema. A diagnosis is confirmed by histopathology. A high index of suspicion can lead to accurate, timely diagnosis and management of the disease.
